# Oral Perceptual Discrimination of Viscosity Differences for Non-Newtonian Liquids in the Nectar- and Honey-Thick Ranges

**DOI:** 10.1007/s00455-014-9518-9

**Published:** 2014-03-30

**Authors:** Catriona M. Steele, David F. James, Sarah Hori, Rebecca C. Polacco, Clemence Yee

**Affiliations:** 1 Toronto Rehabilitation Institute, University Health Network, 550 University Avenue, #12-101, Toronto, ON M5G 2A2 Canada; 2 Institute of Biomaterials and Biomedical Engineering, University of Toronto, Toronto, ON Canada; 3 Bloorview Research Institute, Toronto, ON Canada; 4 Department of Mechanical & Industrial Engineering, University of Toronto, Toronto, ON Canada

**Keywords:** Deglutition, Deglutition disorders, Dysphagia, Sensation, Perception, Viscosity, Discrimination, Oral

## Abstract

Thickened liquids are frequently used in the management of oropharyngeal dysphagia. Previous studies suggest that compression of a liquid bolus between the tongue and the palate in the oral phase of swallowing serves a sensory function, enabling the tuning of motor behavior to match the viscosity of the bolus. However, the field lacks information regarding healthy oral sensory discrimination ability for small differences in liquid viscosity. We undertook to measure oral viscosity discrimination ability for five non-Newtonian xanthan gum-thickened liquids in the nectar- and honey-thick range. Xanthan gum concentration ranged from 0.5 to 0.87 % and increased by an average of 0.1 % between stimuli in the array. This translated to an average apparent viscosity increase of 0.2-fold between adjacent stimuli at 50 reciprocal seconds (/s). A triangle test paradigm was used to study stimulus discrimination in 78 healthy adults in two, sex-balanced age cohorts. Participants were provided 5-ml samples of liquids in sets of three; one liquid differed in xanthan gum concentration from the other two. Participants were required to sample the liquid orally and indicate which sample was perceived to have a different viscosity. A protocol of 20 sets (60 samples) allowed calculation of the minimum difference in xanthan gum concentration detected accurately. On average, participants were able to accurately detect a 0.38-fold increase in xanthan-gum concentration, translating to a 0.67-fold increase in apparent viscosity at 50/s. The data did not suggest the existence of a nonlinear point boundary in apparent viscosity within the range tested. No differences in viscosity discrimination were found between age cohorts or as a function of sex. The data suggest that for xanthan gum-thickened liquids, there may be several increments of detectably different viscosity within the ranges currently proposed for nectar- and honey-thick liquids. If physiological or functional differences in swallowing can be demonstrated for these smaller increments of detectably different viscosity, more narrowly defined categories of thickened liquids for dysphagia management will be warranted.

## Introduction

The use of texture modification has become a cornerstone of dysphagia management [1, 2]. Thickened liquids are frequently recommended to reduce the risk of aspiration that occurs with thin liquids [3]; the primary mechanism of benefit with this intervention appears to be the slower flow of thicker fluids into the pharynx due to their increased viscosity [4]. Despite the widespread use of thickened liquids in clinical practice, the field still lacks clear evidence regarding the degree of thickening required for optimal patient benefit. Consensus-based guidelines in North America (i.e., the National Dysphagia Diet [5, 6]), Australia [7], and the UK [8] concur in classifying liquids in three categories of increasing viscosity. In North America, these categories are commonly labeled as “nectar-thick,” “honey-thick,” and “spoon-thick.” The proposed viscosity boundaries for these categories span quite large ranges of apparent viscosity at a shear rate of 50 reciprocal seconds (50/s), as summarized in Table 1. Information about actual viscosity measurements is typically absent from the packaging or product monographs for commercially pre-prepared thickened liquid products [9], leading clinicians to infer information regarding viscosity from categorical labels. However, prior studies characterizing pre-prepared thickened liquids or liquids prepared according to recipes for adding powdered thickeners suggest that there is considerable variability in the viscosities of products that are served to patients with dysphagia [2, 9–15]. Because viscosity is poorly controlled, the increments of viscosity that have clinical benefit remain poorly defined [16]. In this article, we approach the question of minimally different increments in the viscosity of thickened liquids for dysphagia management from a sensory perspective. The physiological process of swallowing a liquid is thought to require matching of propulsive tongue forces to the perceived flow properties of the bolus. Therefore, we believe it is important to understand how large viscosity differences need to be in order to be perceived or discriminated in the mouth during the oral phase of swallowing. Answering this question will allow future studies to confirm whether measurable changes in swallowing motor behaviors occur across perceivable boundaries and to facilitate new explorations of the clinical benefit of thickening liquids, which incorporate both sensory and motor aspects of swallowing.

**Table 1:** Proposed apparent viscosity ranges for categories of thickened liquid, according to the National Dysphagia Diet [5, 6]

Label	Apparent viscosity (mPa s at 50/s)
Thin	1–50
Nectar-thick	51–350
Honey-thick	351–1,750
Spoon-thick	>1,750

To date, the oral perception of viscosity or liquid thickness has been explored in only a handful of studies. One of the earliest to explore oral rather than nonoral methods of viscosity appraisal [17] showed a linear relationship between perceived viscosity and the actual viscosity of five gum-thickened liquids. This relationship was modeled at a single shear rate to control for shear rate-dependent variations in the viscosity of non-Newtonian liquids. The following year, Christensen [18] reported on a study that used magnitude estimation (i.e., ordered ranking) techniques to explore orally perceived viscosities for aqueous solutions thickened with sodium carboxymethylcellulose. In those experiments participants sampled stimuli and assigned each stimulus a number to indicate its position with respect to the perceived order of progressive thickness for a set of stimuli. A power law exponent of 0.34–0.39 was found to represent the relationship between oral judgments of relative viscosity and physical measurements at a shear rate of 100 reciprocal seconds, with a very strong linear correlation (*r* ^2^ = 0.98). When attempts were made to minimize the influence of nonoral cues on viscosity perception, the power law exponent dropped slightly to 0.29 (*r* ^2^ = 0.88). This result was interpreted to indicate that a tenfold increase in physical viscosity is perceived as an approximate doubling in viscosity. In a subsequent article, Christensen and Casper [19] again used magnitude estimation techniques to rank the perceived viscosity of water thickened with sodium alginate, a gum-based thickener. The power law function for oral perception of viscosity was found to be in a similar range, at 0.39.

More recently, a pair of studies by Smith et al. [20, 21] explored the ability of healthy adult volunteers to accurately rank order the viscosities of Newtonian corn-syrup solutions of increasing viscosity, referenced to anchor stimuli at either end of the continuum. In the first study [20], seven stimuli with viscosities ranging from 3 to 2,240 mPa s at 21 °C were used. In the other study [21], eight stimuli with viscosities ranging from 3 to 2,409 mPa s at 21 °C were studied. Each stimulus in these arrays was either 2.6 [20] or 3 [21] times the viscosity of the preceding stimulus. In the first study [20], participants appraised viscosity during an oral hold task, and identification of rank order was above chance for all pairwise comparisons. In the second study [21], oral holding was compared to actual swallowing of the stimuli, and a power law exponent of 0.33 for oral viscosity perception was found, in strong agreement with the findings of Christensen [18]. Again, it was concluded that the perception of increasing viscosity grows about one fifth as fast as the actual viscosity. In the more recent study by Smith et al. [21], it was found that older participants (over the age of 70) have lower power law exponents, suggesting poorer viscosity discrimination. Smith and colleagues concluded that highly controlled viscosities for clinical use might be unnecessary given that small differences in liquid viscosity were not reliably detected in the mouth. One acknowledged limitation in their study is that by increasing viscosity using greater concentrations of corn syrup, the researchers were unable to avoid the accompanying influence of increased sweetness in judging relative viscosity.

The Newtonian liquids used by Smith et al. [20, 21] included stimuli from both the nectar-thick and honey-thick ranges as defined by the National Dysphagia Diet [5, 6]. Nectar-thick stimuli included viscosities of 82 and 202 mPa s in the first study [20] and 52 and 137 mPa s in the second study [21]. The viscosities of the honey-thick stimuli were 577 mPa s in the first study [20] and 356 and 926 mPa s in the second study [21]. With Newtonian viscosity increments of 2.6- and 3-fold in their studies, it remains unclear whether smaller increments might be perceivable. In the present study, we explored the ability of healthy adult volunteers to perceive differences between five thickened liquid stimuli involving progressive increases in viscosity arising from increases in xanthan gum concentration. The apparent viscosity differences between these stimuli were narrower than those explored previously by Christensen and Casper [19] and by Smith et al. [20, 21]. The decision to use xanthan gum-thickened stimuli was motivated by the fact that xanthan gum has emerged as a commonly used thickener in products developed for the dysphagia market. It is acknowledged that the introduction of either gum or starch thickeners to a liquid results in a liquid with non-Newtonian properties. This means that the apparent viscosity as well as the magnitude of viscosity differences between stimuli varies with shear rate. It is the convention to report the apparent viscosity for non-Newtonian liquids at a shear rate of 50 reciprocal seconds (i.e., 50/s) [6, 22].

Our study was exploratory rather than hypothesis-driven. In contrast to the magnitude estimation protocols used in prior studies, we employed a triangle test paradigm, a robust psychophysical testing protocol for detecting just noticeable differences in sensory stimuli, in which participants are asked to identify one stimulus from a set of three that is perceived to differ from the other two [23–26]. Chance performance for each trial in a triangle test paradigm is 33 %. We use the term apparent viscosity discrimination acuity (AVDA) to refer to a person’s ability to correctly identify liquids with differing xanthan gum concentration and apparent viscosity.

Our objectives were answers to the following questions:

1. What is normal AVDA with non-Newtonian liquids in the nectar- to honey-thick ranges, when tested using a triangle test paradigm?
2. Are there age and/or sex differences in AVDA?

## Methods

### Stimuli

**Fig. 1:**
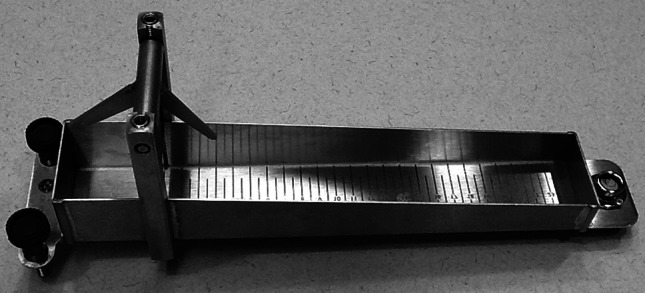
Bostwick consistometer. Flow is measured in this bench-top instrument by releasing a gate between the small containment chamber on the *left side* of the image and observing the distance the liquid flows along the ruler-marked channel on the *right* of the image, given a 30-s flow interval. A spirit level on the far right of the image ensures flow along a level surface

We commissioned an array of liquid stimuli from Flavour Creations Inc. (Brisbane, Australia). In contrast to the stimuli used by Smith et al. [20, 21], our stimuli had identical sucrose concentrations within a flavor (regardless of viscosity). This was done with the hope of avoiding the contaminating influence of sweetness on stimulus discrimination. Four flavors of liquid were provided: cranberry, lime, raspberry, and diet raspberry. The test stimuli were prepared using increasing concentrations (0.5–0.87 %) of a xanthan gum-based thickening agent. The manufacturer custom prepared these test liquids for specified increments of flow, measured at room temperature using a Bostwick consistometer. A Bostwick consistometer is a simple bench device in which a fixed volume of fluid is released from a chamber to flow into an adjacent channel, which is marked to measure the leading edge of the liquid when it comes to rest (see Fig. 1). The unit of Bostwick measurement is expressed as distance flowed (cm) over a 30-s interval. Labeling of thickened fluids using Bostwick units is common for dysphagia products in Australia and some other jurisdictions. However, in comparison to testing using a rheometer, measures made with a Bostwick consistometer are considered crude in rheological circles. For this study, we requested that Flavour Creations Inc. target consecutive, discrete 2-cm increments of Bostwick flow: 10–12, 12–14, 14–16, 16–18, and 18–20 cm. The manufacturer’s certificate of authentication confirmed that the test liquids fell within these requested ranges, based on five repeated tests per batch of liquid at room temperature (18–22 °C), with a test-rest variation of not more than 0.25 cm in flow. The five liquids were labeled A, B, C, D and E, respectively, such that A was the thickest, with a xanthan-gum concentration of 0.87 % and a flow distance between 10 and 12 cm, and E was the thinnest, with a xanthan-gum concentration of 0.5 % and a flow distance between 18 and 20 cm.

**Fig. 2:**
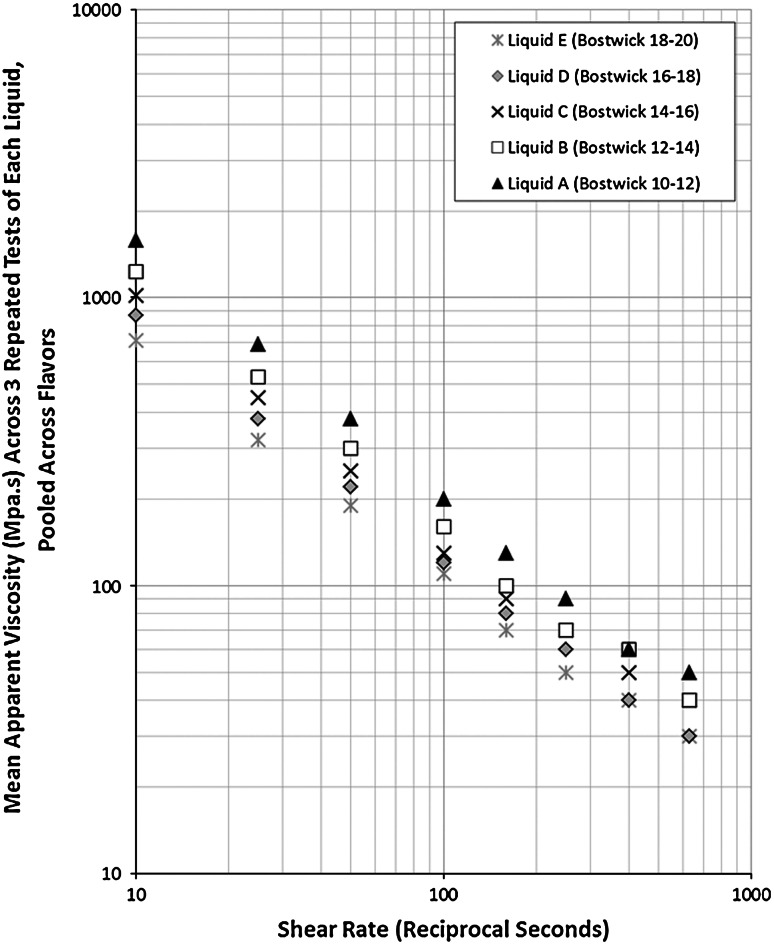
The variation of viscosity across shear rate for the five liquids, pooled across flavor

Upon receipt of these liquids, the viscosity of each one was measured using a TA Instruments AR2000 Advanced Rheometer. We used a cone-and-plate fixture (40 mm, 2°) and carried out bidirectional shear rate sweeps from 0.1 to 1,000/s, with 10 points per decade and a 1-min equilibration period at each end of each sweep. Each sample was tested in triplicate. All tests were performed at 10 °C using the Peltier plate temperature control on the rheometer. A testing temperature of 10 °C was chosen based on recommended serving temperatures for cold beverages in healthcare facilities. These tests revealed no significant differences in viscosity across the four flavors and no significant deviations from the original measurements on repeated testing at 3-month intervals afterward. Figure 2 presents the results of apparent viscosity versus shear rate for the five different stimuli, pooled across flavor. The plot shows that the apparent viscosity measurements for all five liquids maintained their rank-ordered separation over the entire shear rate range, although measures begin to converge, as expected, at higher shear rates.

Table 2 summarizes the differences in xanthan gum concentration and the mean values of apparent viscosity of the five liquids in the stimulus array. Results are pooled across flavor and three repeated sweeps of each stimulus and are reported in mPa s, rounded to the closest unit of 10 for shear rates ranging from 10/s to 630/s. At 50/s, it can be seen that the apparent viscosity of liquid E (the thinnest in our array) was 190 mPa s, falling just below the middle of the National Dysphagia Diet nectar-thick range (Table 1). Liquids B and A, our thickest liquids, had mean apparent viscosities of 370 and 380 mPa s, respectively, falling in the National Dysphagia Diet honey-thick range. Furthermore, it can be noted that liquid A had double the apparent viscosity of liquid E, while the other liquids fell in between, with magnitudes increasing at an average of 0.2-fold (range = 0.14–1.0-fold), as summarized in Table 2. It should be emphasized that even though the spacing of Bostwick measurements was approximately even, the apparent viscosity differences between liquids were not uniform when measured using the rheometer. In all cases, the magnitude of apparent viscosity difference for pairwise comparisons of the test liquids fell below the 2.6- and 3-fold increments explored in prior studies [20, 21]. For the purposes of analysis in this study, we grouped our pairwise stimulus comparisons with respect to the magnitude of differences in xanthan gum concentration as follows:

**Table 2:** Rheological characteristics of the liquid stimulus array used for the experiment (pooled across flavor)

Liquid	% Concentration xanthan	Bostwick	Apparent viscosity (mPa s) at 10/s	Apparent viscosity (mPa s) at 25/s	Apparent viscosity (mPa s) at 50/s	Apparent viscosity (mPa s) at 100/s	Apparent viscosity (mPa s) at 160/s	Apparent viscosity (mPa s) at 250/s	Apparent viscosity (mPa s) at 400/s	Apparent viscosity (mPa s) at 630/s
A	0.87	10:12	1,580	690	380	200	130	90	60	50
B	0.67	12:14	1,230	530	300	160	100	70	60	40
C	0.63	14:16	1,020	450	250	130	90	70	50	40
D	0.58	16:18	870	380	220	120	80	60	40	30
E	0.50	18:20	710	320	190	110	70	50	40	30

Viscosity was measured at 10 °C on a TA instruments AR 2000 advanced rheometer using cone-plate geometry and bidirectional shear rate sweeps from 0.1 to 1,000/s. Values reported represent mean measurements obtained across three repeated sweeps per stimulus and pooled across the four flavors of liquid

- The comparisons of liquids A–B, B–C, B–D, C–D, and D–E had an average difference in xanthan gum concentration of 0.15-fold, corresponding to a mean difference of 0.21-fold in apparent viscosity (range of 0.16–0.26-fold across the shear rate range of 10–630/s). These liquids were grouped together as having a small difference (level 1) in apparent viscosity.
- The comparisons of liquids A versus C and B versus E cluster together, with a mean difference in xanthan gum concentration of 0.36-fold and a corresponding mean apparent viscosity difference of 0.46-fold, which we refer to as a level 2 difference.
- The comparison of liquids A versus D had a difference in xanthan gum concentration of 0.5-fold and an apparent viscosity of 0.66-fold, which we refer to as a level 3 difference.
- The comparison of liquids A versus E had a difference in xanthan gum concentration of 0.74-fold and an apparent viscosity difference of 0.88-fold, which we refer to as a level 4 difference.

The comparison of liquids C versus E, which would have had a level 1 xanthan gum concentration difference of 0.26-fold and an apparent viscosity difference of 0.33-fold, was not included in the data collection protocol.

### Participants

The study sample comprised 78 healthy adults recruited from two age cohorts (“young,” 18–40 years; “mature,” over 60 years). After consent, an intake interview was conducted to confirm eligibility to participate. Participants were asked to review a list of exclusion criteria, as summarized in Table 3, and to disclose whether any of these applied. Disclosure of any one of these resulted in exclusion from the study.

**Table 3:** Exclusion criteria for the study

Medical conditions
• People with a prior medical history of stroke
• People with a prior medical history of acquired brain injury
• People with a diagnosis of Parkinson’s disease
• People with a diagnosis of multiple sclerosis (MS)
• People with a diagnosis of amyotrophic lateral sclerosis (ALS)
• People with a diagnosis of Huntingdon’s disease
• People who have slurred speech or facial muscle problems
• People who have a swallowing disorder
• People who have gastrointestinal problems
• People who have had frequent or chronic sinus infections in the past year
• People who have type I (insulin-dependent) diabetes
• People who have had surgery in the head and neck area (other than tonsillectomy or adenoidectomy)
• People who have had radiation to the head and neck for cancer
• People who experience extreme mouth sensitivity (e.g., when you go to the dentist)
• People who wear a full upper-plate denture, which covers the roof of the mouth, and who are unable/unwilling to remove for the experiment
Medication, drug, and alcohol use
• People who are taking sleeping pills or medication that makes them drowsy
• People who are taking “anti-Parkinson’s” medications like Levodopa
• People who are experiencing dry mouth as a side effect of medication
• People who use drugs (cocaine, methamphetamine, heroin, Ecstasy, etc.)
• People who drink more than two alcoholic drinks per day (i.e., more than social drinking)
• People who currently smoke cigarettes regularly, or have been smokers in the past year
• People who are taking medicine that affects their sense of taste or smell

After confirmation of eligibility, a brief oral mechanism examination was performed by a licensed speech-language pathologist prior to accepting the participant into the study. The final participant sample comprised 21 women and 19 men in the younger cohort, with a mean age of 27 years (range = 18–39), and 22 women and 16 men in the mature cohort, with a mean age of 70 years (range = 60–87). Data collected for one participant were incomplete and therefore discarded. This study protocol was approved by the local institutional research ethics board.

### Triangle Test

**Fig. 3:**
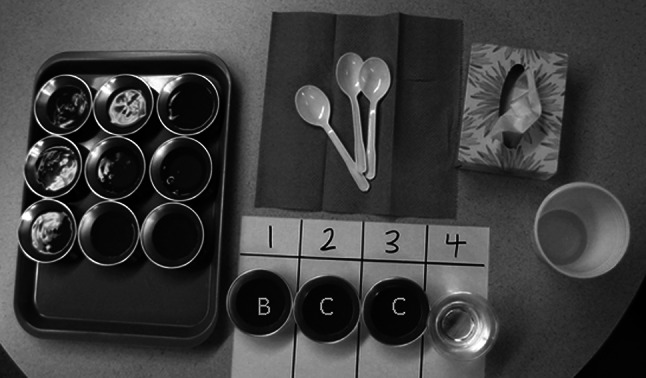
Photo of the experimental setup for the triangle test of viscosity discrimination. Participants were handed teaspoons of liquid for each stimulus in a set, moving from left to right, from the liquids in positions *1*, *2*, and *3* on the template in the *lower right* of the image. They were blinded to the viscosities of the liquids in the set (indicated by the labels *B*, *C*, and *C* in this example) and did not spoon the liquids themselves. One liquid in the set was of a different xanthan-gum concentration and apparent viscosity than the other two. After sampling a liquid and appraising its viscosity orally for 2–3 s, participants were allowed to swallow the sample or expectorate into the white spittoon cup. After sampling all three liquids in a set, participants reported which liquid they had detected as differing in viscosity from the other two. They then used the cup of water in position *4* on the template to rinse before proceeding to a new stimulus set. In total, each participant tested 20 sets

Each participant was seated at a table containing a tray of stimuli. As shown in Fig. 3, the tray contained rows of cups, in sets of three, with each cup containing a sample of test fluid. The test stimuli were organized so that all three cups in each row contained the same flavor of liquid (cranberry, lime, raspberry, or diet raspberry) and had the same color, opacity, and volume. Each participant was randomly assigned to sample stimuli from two of the four flavors during their data collection session. Each set of three stimuli contained examples from two of the stimulus flow levels (A, B, C, D, or E) such that one cup contained a stimulus that was different in xanthan gum concentration from the other two cups in the set. The magnitude of the stimulus difference for each set was randomly assigned using nine pairwise combinations of the stimuli (i.e., A–B, A–C, A–D, A–E, B–C, B–D, B–E, C–D, and D–E). The position of the “different” stimulus in the set was random, and whether it was thicker or thinner was random. Figure 3 illustrates the experimental set up.

To limit potential bias that might arise when spooning or handling the stimuli, for each liquid the experimenter fully loaded a 5-ml teaspoon and handed the spoon to the participant for sampling. The participant was instructed to take the liquid from the spoon into their mouth and to appraise its thickness by pressing the liquid up toward the palate with the tongue over a 2–3-s interval. After appraising the thickness, the participant either swallowed or expectorated the sample. This was repeated for each sample in the set in turn (from left to right). After sampling all three liquids in a set, the participant was asked to report which liquid of the three was perceived to be different in thickness from the other two. In the event that the participant felt that they could not tell which stimulus was different, they were asked to make their best guess. Before proceeding to the next set, the participant rinsed with water. In total, each participant completed 20 sets (60 samples), with the samples organized such that all nine stimulus pairs were experienced at least once and the four levels of xanthan gum concentration difference were each encountered at least twice.

### Data Processing

The participant’s responses (i.e., identification of the liquid that was different) and classification (correct, incorrect) were recorded by a research assistant for each set of stimuli. Response accuracy was logged as a function of the xanthan gum concentration difference magnitude (1, 2, 3, or 4 level) and as a function of stimulus pairing (A–B, A–C, A–D, A–E, B–C, B–D, B–E, C–D, or D–E). The number of correct responses for each magnitude difference level was divided by the number of sets presented to test that magnitude difference, to yield a % correct response rate for each magnitude difference (1, 2, 3, and 4 level). The magnitude of the xanthan gum concentration difference was translated to the corresponding difference in apparent viscosity at 50/s. The average xanthan gum concentration difference magnitude and the corresponding apparent viscosity difference magnitude for correct responses were calculated to yield mean xanthan concentration discrimination acuity (MXCDA) and mean apparent viscosity discrimination acuity (MAVDA) scores. To illustrate, if a participant correctly identified the “different” item in 9 of 20 stimulus sets, with xanthan gum concentration difference magnitudes of 1.26, 1.34, 1.38, 1.50, 1.74, 1.26, 1.34, 1.50, and 1.74 tested in those 9 correctly identified sets, their MXCDA score would have been calculated as:

$$ {\text{MXCDA }} = \, \left[ {\sum \left( {1.26, \, 1.34, \, 1.38, \, 1.50, \, 1.74, \, 1.26, \, 1.34, \, 1.50, \, 1.74} \right)} \right]/9 \, = \, 1.45. $$

Using apparent viscosity measures for these same comparisons at 50/s (see Table 2), the corresponding MAVDA score would be calculated as:

$$ {\text{MAVDA }} = \, \left[ {\sum \left( {1.32, \, 1.58, \, 1.52, \, 1.73, \, 2.00, \, 1.32, \, 1.58, \, 1.73, \, 2.00} \right)} \right]/9 \, = \, 1.64. $$

By contrast, a participant who was able to correctly identify only four of these comparisons (e.g., xanthan gum concentration differences of 1.50, 1.74, 1.38, and 1.74) would have a MXCDA score reflecting the average of only these correctly identified comparisons (i.e., 1.59) and a corresponding MAVDA score of 1.81 at 50/s. Higher MXCDA and MAVDA scores reflect blunter stimulus discrimination.

### Analysis

The frequency of correct responses (i.e., identification of the “different” stimulus) was calculated for the entire data set of 1,540 stimulus trials tested across the 77 participants who successfully completed the protocol. Frequencies were computed separately by stimulus pairing (A–B, A–C, A–D, A–E, B–C, B–D, B–E, C–D, and D–E) and by xanthan gum concentration difference magnitude (1, 2, 3, and 4 level). Pearson’s χ^2^ statistics were calculated to identify significant trends in these distributions. Group mean scores and 95 % confidence intervals for MXCDA after 20 trials and the % correct responses by difference magnitude were calculated overall. Finally, a univariate analysis of variance (ANOVA) was performed to explore cohort, sex, and cohort × sex interaction effects on the dependent variable of MXCDA after 20 trials, with an a priori α criterion set at *p* < 0.05. All statistical analyses were performed using SPSS ver. 22 (SPSS, Inc., Chicago, IL).

## Results

**Fig. 4:**
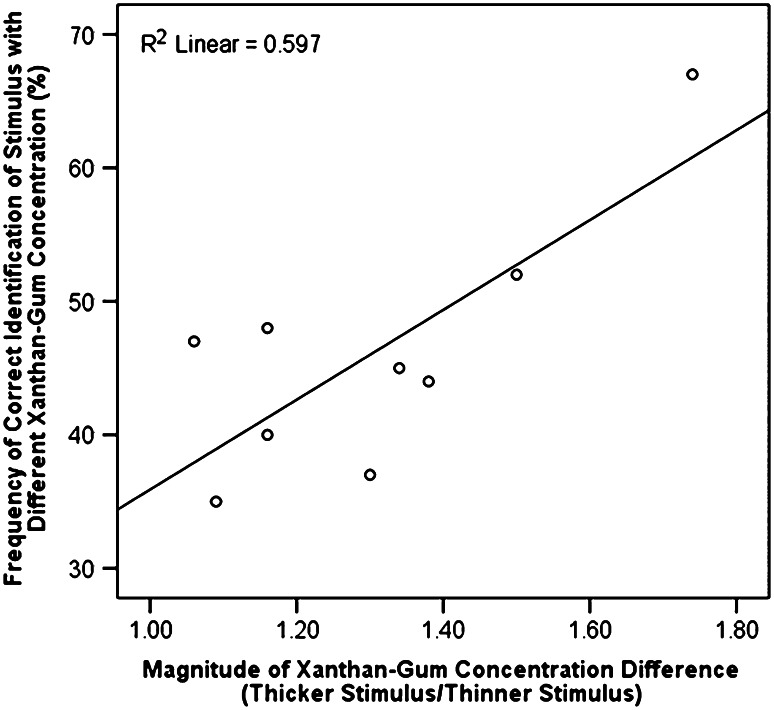
Relationship between response accuracy for identifying a liquid with a differing xanthan gum concentration in a triangle-test paradigm (% accurate) and the magnitude of the difference tested in the stimulus set

Table 4 gives the frequency distribution of correct responses by the magnitude of the xanthan gum concentration difference and stimulus pair over the entire data set. The testing protocol, which included a minimum of two trials per participant for each comparison, showed accurate identification of the liquid with differing xanthan gum concentration, regardless of the stimuli used, at least 43 % of the time. Not surprisingly, the larger 3- and 4-level magnitude differences were detected with greater accuracy (52 and 67 %, respectively) than the 1- and 2-level comparisons (43 and 45 %, respectively). Pearson’s χ^2^ tests revealed the pattern of response accuracy by difference magnitude to be significant (χ^2^ = 41.91, df = 3, *p* = 0.000). χ^2^ tests also identified statistically significant differences in the frequency of accurate response by stimulus pair (χ^2^ = 49.91, df = 8, *p* = 0.000). Of these pairwise comparisons, discrimination of the largest difference (i.e., liquid A vs. E) was the most accurate (i.e., 67 % correct). Figure 4 illustrates the overall relationship found between the frequency of response accuracy and the magnitude of xanthan gum concentration difference for pairwise stimulus comparisons, displaying a statistically significant correlation (Pearson’s *r* = 0.77, *p* = 0.015, *R* ^2^ = 0.6). These results illustrate that performance accuracy for discriminating differences in xanthan gum concentration begins to exceed 0.5-fold when the magnitude of difference is greater than 1.5-fold.

**Table 4:** Frequency distribution of correct identification of apparent viscosity difference by magnitude (level) and stimulus pair

Stimulus pair	Magnitude of xanthan gum concentration difference^a^	Magnitude of apparent viscosity difference at 50/s	% Correct responses by comparison	Level of xanthan gum concentration difference	% Correct responses (by level)
B–C	1.06	1.23	47 %	1	43 %
C–D	1.09	1.08	35 %		
D–E	1.16	1.09	40 %		
B–D	1.16	1.33	48 %		
A–B	1.30	1.25	37 %		
B–E	1.34	1.45	45 %	2	45 %
A–C	1.38	1.54	44 %		
A–D	1.50	1.67	52 %	3	52 %
A–E	1.74	1.82	67 %	4	67 %

^a^The magnitude of difference between stimuli is expressed as the concentration of the thicker liquid divided by the concentration of the thinner liquid

Descriptive statistics (means and 95 % confidence intervals) for MXCDA after completion of all 20 trials are given in Table 5, broken down by cohort and sex. On average, the participants in this study, all of whom were healthy, were able to accurately discriminate a 0.38-fold increase in xanthan-gum concentration, corresponding to a 0.67-fold increase in apparent viscosity at 50/s. It is important to caution that this discrimination resolution may be valid only within the rheological boundaries of the liquids tested, i.e., in the range of 170–400 mPa s (at 50/s) and thus represents a snapshot of a narrow range along the broader continuum for which power law exponents have previously been modeled.

**Table 5:** Descriptive statistics for mean xanthan gum concentration discrimination acuity (MXCDA) after 20 completed discrimination trials using a triangle test paradigm and liquids ranging in xanthan-gum concentration from 0.5 to 0.87 %

Group	Subgroup	Mean	Standard deviation	95 % confidence interval	
				Lower bound	Upper bound
Young	Female	1.39	0.06	1.36	1.41
	Male	1.38	0.06	1.35	1.41
	Cohort total	1.38	0.06	1.37	1.40
Mature	Female	1.37	0.06	1.35	1.40
	Male	1.38	0.05	1.35	1.41
	Cohort total	1.37	0.06	1.36	1.40
Grand mean		1.38	0.07	1.37	1.39

The ANOVA found no significant differences in MXCDA after 20 trials, based on the factors of cohort [*F*(1,73) = 0.36, *p* = 0.55], sex [*F*(1,73) = 0.005, *p* = 0.95], or their interaction [*F*(1,73) = 0.19, *p* = 0.67].

## Discussion

The results of this study must be interpreted in the context of acknowledged limitations. First, the findings represent the perception of differences in apparent viscosity, achieved using different concentrations of xanthan gum. The results may not be generalizable to discrimination of liquids thickened with other agents such as modified corn starch because sensory properties (e.g., density, slipperiness, adhesiveness) may differ from liquid to liquid. Shear-thinning liquids, including those thickened with xanthan gum, are routinely used in the management of dysphagia. It has been argued by previous authors [27] that the shear-thinning properties of these liquids are hypothetically desirable for aiding bolus clearance. However, given that shear rates in the mouth during oral appraisal tasks or swallowing remain unquantified at the present time, the results of this study are probably best understood in terms of the discrimination of differences in xanthan gum concentration. The mapping between these perceived differences in xanthan gum concentration and the corresponding magnitude of differences in apparent viscosity has been estimated in relative terms using the ratio of the thicker liquid’s viscosity divided by the thinner liquid’s viscosity at a shear rate of 50/s.

Our purpose was to determine whether healthy individuals were able to reliably detect small differences in apparent viscosity using a triangle test paradigm involving oral appraisal of the liquid stimuli. Although care was taken to minimize the influence of confounding factors on stimulus discrimination, we cannot entirely eliminate the possibility that cues other than differences in viscosity informed participant response. Certainly, it is true that the stimuli were described by participants to be gel-like and to move easily in the mouth. These comments suggest that the discrimination of differences between stimuli may also have involved awareness of phenomena like the ease of liquid flowing or squirting into the buccal cavity. Similarly, we acknowledge that information may have been gleaned during post-appraisal swallowing or spitting. Further, although efforts were taken to limit the possibility of bias in the handling of the stimuli, we cannot rule out the possibility that participants got clues from the behavior of a stimulus prior to taking it off the spoon. Although a constant sucrose concentration was used across all stimuli, we cannot rule out the possibility that other taste or aroma cues may have played a part in differentiating the test liquids from each other. Given that our understanding of the differences in viscosity across stimuli was based on measurements at 10 °C, it is also plausible that differences in fluid behavior as they warmed to body temperature during the oral appraisal task may have influenced participant response.

In addition to these methodological limitations, we acknowledge that our stimuli were commissioned to fall inside viscosity ranges defined using Bostwick consistometry, which is a crude measurement of fluid flow. As a result, we ended up with a stimulus array that did not include evenly spaced increments of apparent viscosity at 50 reciprocal seconds. The magnitude of detectable difference in apparent viscosity must, consequently, be considered an estimate that arises from the specific magnitudes of difference tested. It would be ideal for future studies to explore more evenly spaced increments in apparent viscosity, similar to the magnitude-estimation methods used in the previous studies by Smith et al. [20, 21]. We also caution that the cognitive, concentration, and memory demands of the triangle test paradigm may not be equivalent to those required in magnitude estimation paradigms; consequently, caution is warranted when comparing results across studies using different methods. Finally, it must be acknowledged that the data represent perceived experience rather than a less overt marker of sensory function.

Notwithstanding these limitations, the current data provide new information regarding the ability of healthy individuals to discriminate small differences in xanthan gum concentration and corresponding differences in apparent liquid viscosity within the range of nectar-thick and honey-thick liquids that are commonly used to address aspiration concerns in individuals with dysphagia. In contrast to previous studies, non-Newtonian liquids with narrower increments of increasing apparent viscosity were studied. The data show that, on average, within the nectar-honey-thick range, healthy adults are able to reliably identify a 0.38-fold increase in xanthan-gum concentration, which translates to a 0.67-fold increase in apparent viscosity. If we extrapolate this result to viscosity ranges beyond those tested in our protocol, we can propose a testable hypothesis that healthy individuals should be able to perceive differences in apparent viscosity between liquids with values of 5, 8, 13, 22, 36, 60, 100, 170, 280, 470, 790, 1,320, and 2,200 mPa s at 50/s. This list of values provides a starting point for future research to confirm the thresholds and magnitude of detectable differences in apparent viscosity for non-Newtonian liquids.

These findings have several interesting implications. First, the findings are in general agreement with those by Smith et al. [20, 21], which showed that healthy adults do not, in fact, have extremely precise viscosity discrimination ability in the mouth. However, we believe our findings do not entirely support Smith’s assertion that precise control of viscosity for clinical purposes may be unnecessary. As would be expected based on power law, our data concur with previous studies in illustrating that small differences in viscosity are detectable at the thinner end of the continuum, but that as viscosity increases, only larger variations are detectable. From a clinical perspective, this raises the interesting possibility that the benefits of very slight thickening (i.e., between 1 and 50 mPa s) may be worth exploring for patients who aspirate thin liquids. On the other hand, our data suggest that the tolerance for variations in viscosity that is currently implied by the labeling system of the National Dysphagia Diet [6] is probably too broad. Our data speak to the fact that healthy individuals likely would be able to perceive at least four grades of increasing apparent viscosity within the 51–350-mPa s range that is currently labeled as nectar-thick. Similarly, the range currently labeled as honey-thick, which spans 351–1,750 mPa s, appears likely to contain at least four discriminable increments of apparent viscosity. Whether such differences in apparent viscosity are clinically relevant and large enough to elicit physiological or functional differences in swallowing (such as reductions in aspiration or differences in the probability of post-swallow residue) remains a question for future research. Until then, our findings point to the probable need for additional subcategories and tighter margins of error in the production of thickened liquids for clinical use.

Interestingly, our results differ from those previously reported by Smith et al. [21] in that we found no evidence of age-related differences in apparent viscosity discrimination. Smith’s oldest participant group had an average age of about 76 years, slightly older than our corresponding group. Notwithstanding the potential confounding influence of increasing sweetness across the stimuli tested by Smith et al. [21], firmer evidence of age-related differences in viscosity discrimination may require exploration of a broader range of viscosities than those tested in our experiment. The absence of an age-related difference in our sample is notable given the heavy cognitive load of the triangle task paradigm. Our results concur with those of Smith et al. [21] in that we found no evidence of sex differences or sex × cohort interactions in oral viscosity perception.

A question that arises from the current experiment is whether the perception of differences in viscosity occurs linearly or logarithmically, as reported previously, or perhaps crosses nonlinear boundaries along the viscosity continuum, i.e., the viscosity may be perceived to have suddenly shifted to being thicker. Unfortunately, the present data are insufficient to clearly answer this question. While it is true that our thickest liquid (A) was most successfully discriminated from the two thinnest liquids (D and E), the pair A–B, which straddles the proposed boundary between nectar- and honey-thick liquids according to the National Dysphagia Diet (Table 1), was not discriminated with markedly different success than other adjacent pairs (B–C, C–D, and D–E). Further exploration with liquids in ranges adjacent to those tested in the current experiment will be needed to elucidate this question. If perceivable nonlinear boundaries in apparent viscosity can be demonstrated in future studies, research to illustrate whether swallowing physiology changes in systematic ways across these boundaries would be warranted, in both healthy individuals and individuals with dysphagia.

## Conclusions

In conclusion, this study adds to the available literature regarding oral perception of differences in apparent viscosity arising from liquids thickened with different concentrations of xanthan gum. The data concur with previous studies in showing that healthy adults do not have terribly fine oral perceptual discrimination of apparent viscosity, at least when tested using a triangle test paradigm that relies on cognitive awareness of perceived differences. Our data suggest that a 0.38-fold increase in xanthan-gum concentration, corresponding to an increase in apparent viscosity of 0.67-fold (at 50/s), should be detectable for non-Newtonian liquids in the nectar- to honey-thick range. Our results also suggest that future research is warranted to confirm whether physiological differences in swallowing behavior or function occur at boundaries along the viscosity continuum that are narrower than those currently proposed for categorizing thickened liquids according to the National Dysphagia Diet [5, 6]. Once the physiological relevance of perceived viscosity boundaries is better understood, the field will be in a better position to develop evidence-based guidelines for target viscosities (and tolerable margins of error around these targets) to guide the production of thickened liquids for use in the clinical assessment and management of dysphagia. Our data suggest that the current categories of thickened liquids defined in the National Dysphagia Diet may encompass clinically relevant subcategories for which apparent viscosity is perceivably different.
